# Kickstarting Immunity in Cold Tumours: Localised Tumour Therapy Combinations With Immune Checkpoint Blockade

**DOI:** 10.3389/fimmu.2021.754436

**Published:** 2021-10-18

**Authors:** Elizabeth Appleton, Jehanne Hassan, Charleen Chan Wah Hak, Nanna Sivamanoharan, Anna Wilkins, Adel Samson, Masahiro Ono, Kevin J. Harrington, Alan Melcher, Erik Wennerberg

**Affiliations:** ^1^ Department of Radiotherapy and Imaging, Institute of Cancer Research (ICR), London, United Kingdom; ^2^ Department of Life Sciences, Imperial College London, London, United Kingdom; ^3^ Leeds Institute of Medical Research at St. James, University of Leeds, Leeds, United Kingdom

**Keywords:** oncolytic virus, radiotherapy, tumor microenvionment, immune checkpoint inhibitors, immunosuppression

## Abstract

Cancer patients with low or absent pre-existing anti-tumour immunity (“cold” tumours) respond poorly to treatment with immune checkpoint inhibitors (ICPI). In order to render these patients susceptible to ICPI, initiation of *de novo* tumour-targeted immune responses is required. This involves triggering of inflammatory signalling, innate immune activation including recruitment and stimulation of dendritic cells (DCs), and ultimately priming of tumour-specific T cells. The ability of tumour localised therapies to trigger these pathways and act as *in situ* tumour vaccines is being increasingly explored, with the aspiration of developing combination strategies with ICPI that could generate long-lasting responses. In this effort, it is crucial to consider how therapy-induced changes in the tumour microenvironment (TME) act both as immune stimulants but also, in some cases, exacerbate immune resistance mechanisms. Increasingly refined immune monitoring in pre-clinical studies and analysis of on-treatment biopsies from clinical trials have provided insight into therapy-induced biomarkers of response, as well as actionable targets for optimal synergy between localised therapies and ICB. Here, we review studies on the immunomodulatory effects of novel and experimental localised therapies, as well as the re-evaluation of established therapies, such as radiotherapy, as immune adjuvants with a focus on ICPI combinations.

## Highlights

- Immune checkpoint inhibitors have revolutionised cancer therapy, however they remain largely ineffective in the treatment of poorly immunogenic “cold” tumours.
- Localised therapies can be used to enhance tumour immunogenicity and overcome resistance to checkpoint blockade, with minimal additive or overlapping side effects.
- Clinical studies to date have yielded mixed results, from negative studies to results that have already changed clinical practice.
- Future directions include novel combinations featuring alternative checkpoints, co-stimulatory agonists and agents that target pathways that may enhance antigenicity. Further considerations include the optimal scheduling of immune-modulatory agents.

## Introduction

The last decade has seen a revolution in the field of immuno-oncology (IO), driven most notably by the approval and clinical implementation of immune checkpoint inhibitor (ICPI) therapy. In work later recognised in the 2018 Nobel Prize in Physiology or Medicine, James Allison and Tasuku Honjo separately identified two pivotal surface receptors that act as negative regulators of the effector T cell (Teff) response, Cytotoxic T Lymphocyte Associated Protein-4 (CTLA-4) (1) and Programmed Cell Death-1 (PD-1) (2), respectively. CTLA-4 is expressed on both effector and regulatory T cells and competes with the co-stimulatory receptor CD28 for shared ligands CD80 and CD86 (3), thereby inhibiting co-stimulatory signals essential for activation. PD-1 is expressed on activated immune cells and inhibits TCR signalling by binding with its ligands Programmed Cell Death-Ligand 1 (PD-L1) or PD-L2 (4). The checkpoints are immune gatekeepers, with receptor-ligand interactions acting to regulate the effector response to pathogens and maintain immune tolerance (3).

These pathways are frequently exploited by tumour cells as a mechanism of immune evasion. Upregulation of PD-L1 on tumour cells, or production of factors that upregulate checkpoint expression on immune cells, leads to exhausted and dysfunctional effector Teff and promotion of regulatory T cells (Treg) within the tumour microenvironment (TME). Monoclonal antibody (mAb) therapy targeting immune checkpoint pathways was shown to be a potent method of anti-cancer T cell re-invigoration, effectively releasing the brakes that are imposed on effector function by checkpoint-mediated immunosuppression. In 2011, the first anti-CTLA-4 mAb Ipilimumab was approved for clinical use, shortly followed by agents targeting the PD-1/PD-L1 axis (5).

Checkpoint inhibitors targeting CTLA-4, PD-1, and PD-L1 now form part of first or second-line standard-of-care in melanoma, non-small cell lung cancer (NSCLC), advanced head and neck squamous cell cancer (HNSCC), renal cancer and urothelial cancer, among others (targets summarised in **Table 1**). The result is a shift in outlook for a subset of patients with previously untreatable cancers (6), and for some a chance of long-term cure. In melanoma, for example, ICPI therapy has seen huge success. The Checkmate 067 trial of dual checkpoint blockade (CTLA-4/PD-1) in advanced melanoma showed a response rate of 58%, with a 52% 5-year survival in a historically poor-prognosis group (7), and patients with a complete response were shown to have a less than 10% chance of relapse on discontinuation of treatment in a study by Robert et al. with a 2-year median follow up (6).

**Table 1:** Current checkpoint inhibitors with regulatory approval.

Target	Checkpoint Inhibitor	Year of first FDA approval
**PD-1**	Pembrolizumab	2014
	Nivolumab	2014
	Cemiplimab	2018
	Dostarlimab	2021
**PD-L1**	Atezolizumab	2016
	Durvalumab	2017
	Avelumab	2017
**CTLA-4**	Ipilimumab	2011

These have undoubtedly been exciting times for the field of IO, and the potential for durable therapy with non-overlapping side-effects continues to bolster clinical and academic interest. Over 3000 clinical trials involving ICPI or other T cell modulators are currently ongoing worldwide. Furthermore, evidence for cancer therapy by inhibition of alternative checkpoints such as T cell and mucin-domain containing-3 (TIM-3), lymphocyte activation gene-3 (LAG-3) and T cell immunoreceptor with immunoglobulin and ITIM domain (TIGIT) is emerging (8).

Although ICPI have revolutionised the treatment landscape, low response rates and resistance still plague effective therapy for the majority. On average, only around 12% of patients gain benefit across all tumour types (9), with particularly low response rates in sites that are seen to be poorly immunogenic such as primary brain, pancreatic and ovarian cancer, and liver metastases (10, 11). Even in sites where ICPI therapy is well established, secondary resistance remains an issue, as seen in the aforementioned landmark Checkmate-067 trial which reported an 11.5-month median duration of response in advanced melanoma (7).

The enduring problem of primary and secondary resistance, combined with a lack of reliable predictive biomarkers of response, leaves a large proportion of patients at risk of ICPI-related toxicity without clinical benefit. A significant focus has therefore been placed on broadening mechanistic understanding of ICPI resistance, and developing strategies to augment response. One strategy is the use of locally-delivered, immune-modulatory therapies in combination with ICPI. These therapies, which include radiotherapy and treatments delivered by intratumoural injection such as oncolytic viruses (OV), can lead to remodelling of the TME to a more favourable phenotype for effective ICPI therapy.

Although accessibility of treatable lesions remains a limitation for some localised therapies, they have several advantages over systemic combinations. They enable targeted manipulation of the TME, minimising off-target effects and systemic or overlapping toxicity. In addition, local treatments have been shown to exert a systemic influence on TME composition and anti-cancer immunity, even in non-treated tumours. Examples of such effects include stimulation of immune cell influx, enhanced immune cell priming and increased expression of checkpoint targets (such as PD-1 or PD-L1). These characteristics form part of the metaphorical notion of the immunological “heat” of a tumour, and are commonly associated with response to ICPI. Accordingly, they are characteristically absent in “cold”, ICPI-refractory tumours. Remodelling of the TME using localised therapies therefore provides potential for global synergy, and enhanced ICPI-responsiveness (12). This review will outline the rationale, pre-clinical and clinical evidence behind localised therapy-ICPI combinations and explore future directions.

### Increasing the Immunological Heat

Immune checkpoints form only part of the complex picture of effective anti-cancer immunity. Huge diversity in mutational burden, antigen release and presentation, inflammatory signalling and TME composition all play a critical role in ICPI efficacy (13). This diversity and resultant dichotomy in ICPI response is apparent between patients of the same tumour type, and even within homogenous mouse tumour models (14).

The unifying concept of tumour Immunological “heat” is a global representation of this multifactorial diversity and represents the ability of a tumour to elicit effective anti-cancer immunity.

Immunostimulatory “hot” tumours are seen to be more responsive to immunotherapy. “Hot” characteristics include an immune-cell rich TME, high in CD8^+^ Teff cells with a high CD8:Treg ratio, antigen-presenting cell (APC) and inflammatory M1-polarised macrophage infiltration, and immune-stimulatory cytokine production (such as Type I IFN). High tumour antigen (TAA) availability due to a high tumour mutational burden (TMB) or microsatellite instability (MSI) has also been associated with ICPI responsiveness, and checkpoint expression on activated, antigen-exposed immune cells, along with PD-L1 on tumour cells, provide targets for ICPI therapy (12).

**Figure 1:**
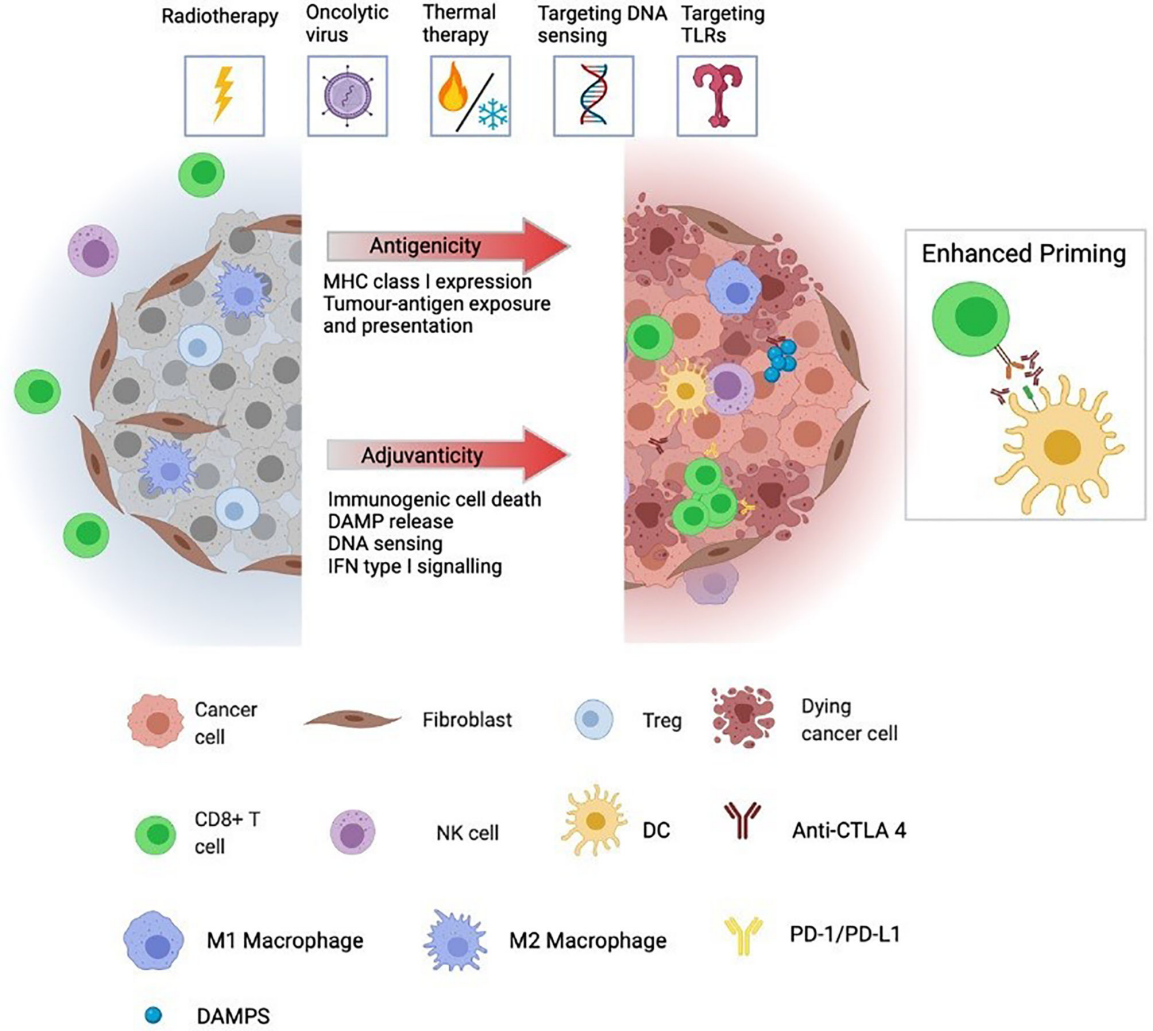
Immunologically “cold” tumours are generally unresponsive to ICPI and characterised by low infiltration and/or exclusion of cytotoxic lymphocytes, including CD8 T cells and NK cells. Further, cold tumours often have high infiltration of immunosuppressive cells including Tregs, CAFs, and M2-polarized macrophages as well as low expression and presentation of tumour neoantigens preventing priming of de novo immune responses. Immunogenic localised therapies are designed to convert ‘cold’ tumours to a ‘hot’ by altering the adjuvanticity and antigenicity of the TME. Antigenicity is achieved by augmented expression, degradation and presentation of tumour neoantigens while adjuvanticity is associated with elevated levels of DAMPs, released from dying tumour cells, cytosolic DNA accumulation and sensing, and a transcriptional profile geared towards IFN type I signalling. Together, these factors promote recruitment, infiltration and activation of DCs allowing for increased antigen cross-presentation and priming of tumor-specific CD8 T cells. Triggering of these events by localised therapies creates a favourable environment for synergy with ICPI. The pool of activated cross-presenting DCs cooperates with anti-CTLA-4 treatment to generate a broadened repertoire of tumour neoantigen-specific T cells whose effector function can be augmented by anti-PD-1 treatment in their killing of tumour cells locally and systemically. TME, tumour microenvironment; DAMP, danger-associated molecular pattern; DC, dendritic cell; CAF, cancer-associated fibroblast; ICPI, immune checkpoint inhibitor; NK cell, natural killer cell.

Immunosuppressive “cold” tumours are seen to be less responsive to immunotherapy. They feature absent or excluded Teff, with a higher proportion of immunosuppressive Treg and M2 polarised macrophages. Poor antigen availability, for example in tumours with a low TMB and excluded APCs, means immune cell priming is suboptimal. This inhibits an effective anti-cancer immune response and renders ICPI therapy ineffective or even detrimental. For example, PD-1 blockade has been shown to drive T cell dysfunction and anti-PD-1 resistance in the absence of effective priming (15). A non-reactive gene signature and immunosuppressive cytokine production [such as IL10 and transforming growth factor β (TGF-β)] maintain a paucity of immune cells, and the immune-inhibitory effects of other TME constituents may predominate, for example cancer-associated fibroblasts (CAFs), hypoxia and abnormal vasculature (**Figure 1**) (16).

Although complex, and by no means universal, where a tumour sits on the axis of immunological heat is known to play a pivotal role in the response to ICPI therapy. Resistance mechanisms can remain dominant at the level of the TME even when circulating antigen-specific T cells are high (17), and local manipulation of the TME to increase the “heat” and improve ICPI responsiveness therefore presents a rational therapeutic strategy. Localised therapy/ICPI combinations involving radiotherapy or oncolytic virotherapy have gained the most clinical momentum to date. Further strategies in clinical development include agonists of immune-stimulatory pathways such as Stimulatory of Interferon Genes (STING) and Toll-Like Receptor (TLR) signalling, or physical modification or the TME using thermal treatments, such as high-intensity focused ultrasound (HIFU) or photothermaltherapy (18). This review will outline the effects of these treatments on TME composition and immunogenicity in “cold” tumours, and explore the evidence behind their combination with ICPI therapy.

### Cellular and Molecular Mechanisms Underlying Immune Activation

Despite distinct differences in their mechanisms of action, the localised therapies featured in this review share some commonality in the basic immune-modulatory pathways through which they exert their immune effects and enhance ICPI therapy (summarised in **Table 2**). Advances in immunology research have validated radiotherapy-induced DNA damage as a viral mimic (34), triggering the same intrinsic anti-viral inflammatory pathways that are naturally stimulated by OV therapy (35). These protective pathways can also be targeted downstream by other agents such as TLR or STING agonists, and form the cellular machinery that enable recognition and presentation of pathogenic material or cellular defects - leading to an inflammatory signalling cascade and an innate and adaptive immune response.

**Table 2:** Summary of key mechanisms of therapeutic synergy between localised therapy combinations and ICPI.

Therapy-induced mechanisms	Immunogenic effects promoting synergy with ICPI	References
**Nucleic acid sensing**	Induced IFN type I signalling	(19–23)
cGAS/STING activation	→ T cell recruitment	
RIG-I/MAVS activation	→ Augmented CD8 T cell cytotoxicity	
	→ Increased DC cross-priming	
**DAMP release/exposure**		(24–26)
ATP	Recruitment and activation of DCs	
HMGB1	Increased phagocytosis	
CALR	Production of pro-inflammatory cytokines	
**Neo-antigen expression and processing**	Increased peptide pool	(27–31)
	Increased diversity of TCR repertoire	
	Generation of tumour specific T cells	
**MHC class I upregulation**	Augmented CD8 T cell priming	(27)
	Enhanced tumour cell killing	
**Death-receptor upregulation**	Augmented NK cell and CD8 T cell cytotoxicity	(32, 33)

Pattern-recognition receptors (PRRs) expressed on innate immune cells have evolved to detect microbial pathogenic molecules collectively known as pathogen-associated molecular patterns (PAMPs). The cytosolic nucleic acid sensors cyclic GMP-AMP synthase (cGAS) and retinoic acid inducible gene I (RIG-I) are not only important for detection of infected cells but also for immune recognition of cancer cells (36). Changes in the composition and abundance of cytosolic double-stranded DNA (dsDNA) and dsRNA induced during tumorigenesis, or by cellular stress following therapy, are detected by PRRs such as cGAS and RIG-I respectively, resulting in activation of STING and mitochondrial antiviral-signalling protein (MAVS). The resulting complex downstream signalling, including IRF3 and NFkB-dependent pathways, ultimately leads to expression of type I interferons (IFNs) and other pro-inflammatory cytokines (37, 38).

Two decades ago, Polly Matzinger postulated that immune activation can also occur in the absence of microbial products, instead being triggered by inflammatory signals released from stressed or dying cells (39), which are collectively named damage-associated molecular patterns (DAMPs). DAMPs such as ATP, HMGB1 and calreticulin are hallmarks of the highly inflammatory process of immunogenic cell death (ICD), which is defined as a regulated cell death mechanism capable of inducing an adaptive immune response in the host. Release of the metabolic mediator ATP into the extracellular space triggers recruitment and activation of DCs *via* P2Y2 and P2X7 receptors respectively (40, 41), while secretion of HMGB1 activates DCs *via* TLR-4 (42). Translocation of calreticulin to the cell surface provides an “eat-me” signal to antigen-presenting cells and results in phagocytosis of the target cell (43). In the context of cancer, ICD leads to release of tumour-associated antigens (TAA) and subsequent priming of a cancer-specific immune response.

Together, therapy-induced inflammatory PAMP and DAMP signalling generate a favourable environment for activated DCs to process and cross-present tumour-derived antigens to naïve T cells, which can prime and sustain a systemic tumour-specific immune response in synergy with ICPI. Induction of ICD, and the resultant increase in adjuvanticity of the tumour, is therefore a key mechanism underlying the efficacy of immunogenic localised therapies such as OV and radiotherapy.

## Oncolytic Virotherapy

Oncolytic viruses (OV) are naturally-occurring or genetically-modified (GM) viruses that selectively infect and destroy tumour cells through direct cell lysis and stimulation of an anti-cancer immune response (44). Many tumour cells are intrinsically sensitive to viral infection due to common deficiencies in key anti-viral machinery that enables unhindered viral replication while normal tissue is spared (45), a characteristic that can be optimized for safety and selectivity through variant selection or viral genetic modification.

The immune stimulatory effects of OV are multi-modal. Viral replication triggers cell lysis and ICD. This releases viral progeny to continue the lytic cascade in surrounding tumour cells, as well as TAA for cross-priming of APCs and DAMPs, subsequently leading to stimulation of a Type 1 IFN-mediated anti-tumour immune response (46). The cell intrinsic anti-viral apparatus also plays an integral role in OV-mediated immunity. Viral DNA and RNA are sensed by PRRs such as cGAS and RIG-I respectively, triggering an ATP-dependent inflammatory cascade mediated by STING, leading to JAK/STAT pathway upregulation and pro-inflammatory cytokine release (47).

The result is a switch to an immune-stimulatory TME, with influx of activated T cells and APCs, upregulation of MHC and co-stimulatory markers such as CD40, CD80 and CD86 (48), as well as enhanced antigen presentation. This leads to the upregulation of PD-1 and CTLA-4 by T cells, potentiating immune checkpoint inhibition. This OV-mediated immune-stmulation also presents a barrier to effective OV monotherapy, mediating adaptive resistance and leading to exhausted Teff and Treg influx. Synergy between ICPI and OV herefore has the potential to work both ways; OV may enhance response to ICPI, and conversely ICPI may enhance the efficacy of OV.

In 2015, the oncolytic Herpes Simplex Virus (oHSV), talimogene laherparepvec, became the first OV to gain regulatory approval for cancer therapy (49), leading to an acceleration in OV research. Since this milestone, evidence for the widespread clinical implementation of OV monotherapy has been limited. The 26% ORR and 23.3-month median OS seen with T-Vec in advanced melanoma was surpassed by dual checkpoint blockade, and to date no further OV have gained FDA approval.

What has become apparent is the potential of OV therapy as an immune adjuvant in combination with other immune-modulatory therapies, such as ICPI. In combination, OV present an appealing prospect. They exhibit anti-cancer activity and tumour selectivity, are generally well-tolerated with non-overlapping side-effects, and have the ability to increase the immunological heat of OV-injected and non-injected tumours – a phenomenon demonstrated in both pre-clinical animal models and patients (50).

A further advantage of OV therapy is the application of OV as viral vectors. The large backbone of some OV, such as oncolytic Herpes Simplex Virus (oHSV), Adenovirus (oADV) or Vaccinia Virus (oVV) can be manipulated by insertion of therapeutic transgenes, thus exploiting selective viral replication for concentrated delivery of immune-modulatory agents within the TME. This provides a unique opportunity, not only to manipulate the TME to enhance ICPI therapy, but to deliver the ICPI themselves. This is a particular advantage when considering the delivery of molecules where systemic administration may be limited by toxicity or pharmacokinetic considerations. Examples include the anti-CTLA-4 mAb, or potent immune-stimulators such as agonists of the 4-1BB co-stimulatory receptor or stimulatory cytokine IL-12.

Several clinical trials of OV/ICPI combinations are currently ongoing or have recently been completed, backed by pre-clinical evidence of synergistic effects. This section will focus on the rationale and evidence behind locally-delivered OV and ICPI therapy combinations; strategies featuring systemic OV delivery are reviewed in detail elsewhere (51).

### Localised OV and Checkpoint Blockade Combinations – Pre-Clinical Studies

Extensive pre-clinical research has evaluated the mechanisms behind OV remodelling of the TME in “cold” tumours, and the implications for subsequent checkpoint blockade. Among the most clinically advanced OV to date are variants of the oHSV and oADV viral platforms, double-stranded DNA viruses that are not only highly immune-stimulatory, but have large viral backbones that provide opportunity for transgene insertion.

Zhang et al. showed that oHSV therapy led to an increase in tumour-infiltrating CD4 and CD8 T cells and a decrease in Treg and suppressive TAM in a mouse model of pancreatic ductal adenocarcinoma (PDAC). PDAC is a notoriously immune-excluded, “cold” tumour, with a TME comprising immunosuppressive Treg, tumour associated macrophages (TAM), immunosuppressive cytokines and physical barriers to T cell infiltration such as CAFs and a desmoplastic stroma (52). Transcriptome profiling of immune cells following treatment showed enrichment of PD-1, LAG-3 and TIM-3 in the CD8 T cell population, and OX40 and CTLA-4 in the CD4 population (53). Accordingly, triple combination therapy (OV/PD-1/CTLA-4) was shown to significantly prolong survival in PDAC tumour-bearing mice.

HF-10 (Canepaturev, CRev) is a further oHSV1 which contains natural mutations that enhance selectivity. HF-10 treatment led to an influx of CD8 T cells in a poorly immunogenic HNSCC model, with infiltration of PD-L1-expressing macrophages and DCs in both OV-injected and non-injected tumours. Despite a PD-L1-enriched TME, a therapeutic effect was seen with single-agent HF-10 treatment; however, this was significantly enhanced by addition of anti-PD-L1 therapy. Interestingly, synergy was seen with high-dose, but not low-dose, anti-PD-L1 therapy highlighting a dose-dependent factor in the ability of ICPI to overcome either the intrinsic tumour-mediated immunosuppression or OV-induced checkpoint upregulation (54).

Saha et al. showed enhanced CD8 T cell infiltration in an 005-GSC-derived GBM mouse model following treatment with a third-generation triple-mutated oHSV encoding the immunostimulatory cytokine IL-12 (G47ΔV-mIL-12) (55). Glioblastoma multiforme (GBM) is a highly immunosuppressive tumour, with low response rates to single and dual checkpoint blockade and added immuno-therapeutic complexity provided by the blood-brain barrier and tissue hypoxia (55). In addition to Teff infiltration, changes were seen in other TME cell compartments, with a decrease in the proportion of Treg and an increase in the CD8 T cell/Treg ratio. A shift was seen towards pro-inflammatory M1-polarised macrophages with an increase in IFNy production, indicating a more-immunogenic tumour phenotype. Modest synergy was seen when G47ΔV-mIL-12 was combined with single agent ICPI (PD-1 or CTLA-4), however triple therapy (OV/PD-1/CTLA-4) led to long-term cures and protection from tumour re-challenge. This treatment effect was dependent on CD4 and CD8 T cells, as well as macrophages, highlighting the complex relationship between constituents of the TME, and the potential need for multi-targeted therapy to overcome tumour-mediated immunosuppression.

Adenovirus is a double-stranded DNA virus which has again been extensively investigated in the context of OV therapy, including for GBM. Stereotactic administration of low dose oADV was shown to upregulate PD-1 expression on tumour-infiltrating CD8 T cells, highlighting a mechanism of adaptive resistance. Synergy was seen with anti-PD-1 therapy, with significantly improved survival in GBM tumour-bearing mice (56).

Evidence of efficacy of an oADV encoding co-stimulatory ligand CD40-L was shown by Singh et al. in a mouse melanoma model. Melanoma has been well-established as an immunogenic “hot” tumour site. Despite this, 50% of patients do not respond to dual checkpoint blockade. The B16 mouse melanoma model is highly immunosuppressive, with an immune-excluded TME and production of immunosuppressive cytokines such as TGF-β. Remodelling of the TME was seen following treatment, with an influx of IFNy-producing CD8 T cells, an increase in the CD8:Treg ratio and upregulation of PD-L1 on tumour tissue. Combination treatment with anti-PD-L1 enhanced therapy and led to an increase in CTLA-4-expressing CD8 T cells. Subsequent triple therapy (OV/PD-L1/CTLA-4) significantly improved response, leading to regression of OV-injected and non-injected lesions (including brain metastases). A 45% cure rate was achieved, with protection from tumour re-challenge (57). Hu et al. also demonstrated synergy between a modified oADV, this time armed with immunostimulatory cytokine IL-24, and PD-1 blockade in B16-melanoma tumour-bearing mice. Treatment increased CD8 T cells, Tregs and CD11b+ myeloid cells, with MHC upregulation on APCs and production of inflammatory cytokines (58). Interestingly, although anti-tumour immunity was seen to be dependent on viral attachment and entry, the oADV did not successfuly infect and lyse cells and, therefore, lead to PAMP/DAMP release, and hence did not induce anti-tumour immunity through these mechanisms. Instead, oADV treatment appeared to label tumour cells as “non-self”, leading to enhanced MHC-1 and co-stimulatory CD80 expression, and presentation of “non-self” viral epitopes on the tumour-cell surface, triggering an anti-cancer immune response. This highlights the complex mechanisms surrounding the immune-stimulatory effects of OV (59).

The vaccinia poxvirus, historically used as a vaccine for smallpox since the late 19^th^ century, is also in clinical development as an OV. The modified oVV pexastimogene devacirepvec (JX-594) has yielded disappointing results in clinical trials to date, most notably with the failure of the phase 3 PHOCUS trial in liver cancer (60). However, it has shown pre-clinical promise in combination with ICPI therapy. Remodelling of the TME was demonstrated by Chon et al. following IT JX-594 therapy, with an influx of CD8 T cells and NK cells, upregulation of inflammatory genes and a switch to an immune-stimulatory TME in mouse breast and renal cancer models. As with other studies, combinaton with single-agent ICPI enhanced therapy, however triple therapy (OV/PD-1/CTLA-4) led to complete, durable tumour regression (61). Similar trends were seen in other studies of poorly immunogenic mouse colon and ovarian cancer models (62, 63).

An alternative therapeutic strategy is using OV as viral vectors to deliver ICPI therapy through insertion of transgenes encoding checkpoint blocking antibodies, an attractive prospect when considering complex immunomodulatory combinations and avoidance of systemic ICPI toxicity. Having said that, encoding ICPI or immune activating ligands within OV may present some limitations, not least of all the fact that the two components of therapy are obligatorily expressed within the same tissue compartment, which may not always be the optimum means of combination. Kleinpeter et al. showed that insertion of murine anti-PD-1 into an oVV backbone enhanced therapeutic effects in a poorly-immunogenic mouse fibrosarcoma model (64). Therapy led to a higher and more prolonged intra-tumoural anti-PD-1 concentration than IT injection of the antibody itself, highlighting the advantage of viral replication in dose amplification. Blood levels of anti-CTLA-4 mAb were also shown to be low following IT delivery of the GM oVV BT001 encoding anti-CTLA-4 and GM-CSF, while IT levels were sufficient to suppress CTLA-4 receptor function for days to weeks following injection (65).

Additional pre-clinical evidence was presented by Zuo et al. Up to 70% complete and durable tumour regression was seen in mouse tumour models following treatment with an oVV encoding novel checkpoint TIGIT, which is highly expressed on natural killer T cells (NKT) and Tregs. Treatment stimulated a CD8 T cell-mediated anti-tumour response with evidence of immune memory and protection from re-challenge. High levels of anti-TIGIT mAb were seen in tumour tissue, but not in blood from treated mice (66). A TK-gene deleted oVV expressing anti-PD-1 and an anti-4-1BB co-stimulatory receptor agonist was also shown to suppress tumour growth in mouse models of liver and pancreatic cancer (67).

The oHSV platform is also amenable to genetic modification. Coffin et al. developed the RP oHSV platform, featuring ICP34.5 and ICP47 deletions to attenuate neurovirulence and enhance antigen presentation and GALV-GP-R and hGM-CSF insertion to enhance OV-induced ICD. Further modification with insertion of ICPI or co-stimulatory ligands (CTLA-4, 4-1BB, CD40-L and OX40-L) was shown to increase therapeutic efficacy in tumour-bearing mice (68). Enhanced therapy, this time with an anti-PD-1 armed oADV was also demonstrated by Zhang et al. The un-modified oADV was shown to increase TME immune infiltration and promote PD-L1 upregulation but failed to prolong survival. Genetic modification with the addition of the extracellular domains of PD-1 and CD137L (4-1BBL) led to a 70% long-term cure rate in a sub-cutaneous hepatocellular carcinoma (HCC) model (69). A further modified oADV, LOAd703, also encodes 4-1BBL, along with TMZ-CD40-L, and has shown pre-clinical activity in PDAC mouse models (70).

Other OV in clinical development include polioviruses, Newcastle disease virus (NDV), reovirus and maraba virus. Clinical trials involving both reovirus and NDV in combination with ICPI therapy are ongoing, with pre-clinical evidence of synergy (3, 49, 71, 72); however, these are focused on systemic delivery, and as such are reviewed elsewhere. The non-neurovirulent rhinovirus:poliovirus chimera PVSRIPO was shown to synergise with anti-PD-1 and anti-PD-L1 therapy in mouse triple-negative breast cancer (TNBC) models (73) and is currently the subject of early-phase clinical trials. Maraba virus is a member of the Rhabdovirus family of RNA viruses, and was shown to increase Treg and PD-L1 expression when given prior to tumour resection in mouse breast cancer models. Post-operative addition of dual ICPI therapy (CTLA-4/PD-1) was shown significantly to prolong survival when compared to virus or ICPI alone (74).

## Clinical Translation

### Herpes Simplex Virus

Oncolytic viruses based on the Herpes Simplex Virus-1 (oHSV-1) have gained the most clinical traction to date following the approval of T-Vec in the treatment of melanoma. In T-Vec, the HSV backbone has been modified by deletion of ICP34.5 and ICP47 and insertion of GM-CSF to enhance selectivity and immune effects. T-vec was shown to induce antigen-specific local and systemic immunity in phase II studies, with an increase in CD8 T cell density in injected and non-injected lesions, increased checkpoint expression (50), and an increase in melanoma antigen-specific T cells (75).

T-Vec/ICPI combination therapy has to date yielded mixed results. A phase II trial of IT Tvec and Ipilimumab therapy in advanced melanoma showed a significant improvement in response (38% vs 18%) with regression of non-injected lesions and no additional safety concerns (76). However, a phase III study evaluating T-Vec in combination with Pembrolizumab was recently terminated due to futility at interim analysis (77), despite promising translational data in the phase 1b part of the the trial (78). The MASTERKEY-232 phase Ib study evaluated Tvec in combination with Pembrolizumab in recurrent or metastatic HNSCC. PFS and OS was comparable to documented results of Pembrolizumab monotherapy, and phase III was not pursued (79). As is a common IO theme, impressive and durable responses are seen for a minority. For example, Khaddour et al. reported a case of complete, durable tumour regression in a patient with melanoma with brain metastases following T -Vec, Atezolizumab and Temozolomide therapy (80). This highlights that there is much still to learn about the biology underpinning these and future complex combinatorial strategies. Several trials featuring T-Vec/ICPI combinations are ongoing (**Table 3**).

**Table 3:** Summary of ongoing clinical trials evaluating oncolytic virotherapy in combination with immune checkpoint inhibition.

Oncolytic virus and NCT number	Combination	Status
Herpes Simplex Virus-1 (HSV-1)		
NCT04185311	T-Vec + Ipilimumab + Nivolumab	Active, not recruiting. Phase 1. Neo-adjuvant, breast cancer (TNBC, ER+HER2-)
NCT03842943	T-Vec + Pembrolizumab	Recruiting. Phase 2, neo-adjuvant, stage 3 resectable melanoma
NCT04068181	T-Vec + Pembrolizumab	Active, not recruiting. Phase 2, metastatic melanoma following progression on anti-PD1 therapy
NCT03069378	T-Vec + Pembrolizumab	Recruiting. Metastatic/locally advanced sarcoma
NCT02509507	T-Vec + Pembrolizumab	Recruiting, phase 1b/2. Liver tumours (HCC and liver metastases)
NCT04050436	RP1 + Cemiplimab	Recruiting
		Phase II
		Locally advanced or metastatic cutaneous SCC (CSCC)
NCT03767348	RP1 + Nivolumab	Recruiting
		Phase 1/2
		Advanced and/or refractory solid tumours
NCT04336241	RP2 + Nivolumab	Recruiting
		Phase 1, advanced solid tumours
NCT04735978	RP3 + Nivolumab	Recruiting
		Phase 1, advanced solid tumours
NCT04348916	ONCR-177 + Pembrolizumab	Recruiting. Phase 1, advanced solid tumours and liver metastases
Adenovirus		
NCT04387461	Intravesical CG0070 + Pembrolizumab	Recruiting
		Phase 2, non-muscle invasive bladder cancer
NCT02636036	Enadenotucirev + Nivolumab	Active, not recruiting
		Phase 1, metastatic or advanced epithelial tumours
NCT02798406	DNX-2401 + Pembrolizumab	Active, not recruiting
		Phase 2, glioblastoma and gliosarcoma
NCT04123470	LOAd703 + Atezolizumab	Recruiting
		Phase 1/2, Metastatic melanoma
NCT02705196	LOAd703 + Atezolizumab + standard of care (Gemcitabine/nab-Paclitaxel)	Recruiting. Phase 1/2. Pancreatic cancer.
NCT03172819	OBP-301 + Pembrolizumab	Active, not recruiting
		Phase 1, advanced or metastatic solid tumours
NCT03921021	OBP-301 + Pembrolizumab	Recruiting
		Phase 2, esophagogastric adenocarcinoma
NCT03003676	ONCOS 102 + Pembrolizumab	Active, not recruiting. Phase 1, advanced melanoma after progression on anti-PD-1 therapy
NCT02963831	ONCOS 102 (intraperitoneal) + Durvalumab	Recruiting, phase II
Vaccinia virus		
NCT03294083	Pexa-Vec (JX-594) + Cemiplimab	Recruiting, phase 1b/2a, metastatic or unresectable RCC
NCT02977156	Pexa-Vec (JX-594) + Ipilimumab	Recruiting, phase 1, advanced solid tumours
Poliovirus		
NCT04577807	PVSRIPO + Nivolumab	Phase 2. Advanced, PD1 refractory melanoma
NCT03973879	PVSRIPO + Atezolizumab	Withdrawn (resubmission planned), phase 1/2 glioma
VSV		
NCT02923466	VSV-OFNb-NIS + Avelumab	Active, not recruiting. Phase 1, refractory solid tumours

The spontaneous oHSV mutant HF-10 has also been evaluated in clinical trials in combination with ICPI therapy. A phase II study of HF-10 and Ipilimumab in advanced melanoma demonstrated an acceptable safety profile with a best overall response rate (BORR) of 41% and 19-month median PFS (81). Responding patients had influx of CD4 and CD8 T cells, along with increased CD4 ICOS expression and PD-L1 upregulation on monocytes. HF-10 was also evaluated in combination with Ipilimumab in patients with treatment-refractory acral and mucosal melanoma with an 11.1% BORR and 55.5% disease control rate (82).

The RP oHSV platform developed by Coffin et al. (RP1, RP2 and RP3) is undergoing clinical evaluation, with ongoing phase I and II trials in combination with anti-PD-1 or anti-PD-L1 agents in advanced solid tumours (**Table 3**). An oncolytic HSV-2 virus was also evaluated in a first-in-human phase 1b study in combination with anti-PD-1 therapy in metastatic oesophageal and rectal cancer patients. Remodelling of the TME was apparent, with CD8 T cell infiltration and increased PD-L1 expression, along with evidence of regression of both injected and non-injected lesions (52).

### Adenovirus

Several oADV are in clinical development and have been tested in combination with ICPI therapy. ONCOS-102 is an oADV with a 24bp deletion in the E1A Rb binding site to attenuate replication in normal tissue, and addition of GM-CSF for immune augmentation. A two-part phase I study of ONCOS-102 in combination with concurrent or sequential anti-PD-1 therapy provided evidence of the ability of OV to overcome ICPI resistance. This trial recruited advanced melanoma patients that were refractory to prior anti-PD-1 therapy, and reported a 35% ORR in an early analysis (NCT03003676). A phase I study of ONCOS-102 in combination with Durvalumab in ovarian and colorectal cancer with peritoneal metastases showed an increase in CD8 T cell infiltration and PD-L1 expression following treatment. Some evidence of clinical activity was seen, but only 1 durable response. Phase II recruitment is ongoing (NCT02963831). DNX-2401 is a further oADV with E1A deletion. A phase II dose escalation study of IT DNX-2401 in combination with Pembrolizumab in recurrent GBM noted an 11.9% ORR with 2 ongoing durable responses in the 49 patients recruited, median OS was 12.5 months (83). CG0070 is an oADV armed with GM-CSF. A phase II study evaluating intra-vesical CG0070 in combination with pembrolizumab in immunotherapy-refractory non-muscle-invasive bladder cancer (NMIBC) is ongoing (NCT04387461).

LOAd703 is a modified oADV armed with immune-stimulatory transgenes TMZ-CD40L and 4-1BBL. A phase 1/2 trial is currently recruiting and will evaluate LOAd703 in combination with standard of care chemotherapy (Gemcitabine/nab-Paclitaxel) and Atezolizumab in PDAC (NCT02705196). Pancreatic cancer is notoriously immune-excluded, however combination treatment was shown to increase antigen-specific T cells and reduce circulating MDSCs, with a partial response in 6/10 subjects in an interim report (84). Finally, the modified oADV TILT-123 encodes two immunostimulatory cytokines (IL2 and TNFa) with promising pre-clinical activity in combination with anti-PD-L1 therapy. A study combining TILT-123 with anti-PD-L1 agent Avelumab is planned for 2021.

### Other Locally-Delivered OV in Clinical Development

Synergy has been demonstrated between oncolytic Coxsackie viral strain CVA21 (Cavatek) and ICPI therapy and, as with other OV, added toxicity in combination was minimal. Changes within the TME were seen following CVA21 treatment, with increased CD8 T cell infiltration and upregulation of PD-L1 and other immune checkpoint receptors. The phase Ib MITCI trial evaluated IT CVA21 therapy in combination with Ipilimumab in patients with advanced melanoma. An ORR of 38% was observed with no dose-limiting toxicity (85) (NCT02307149). Interim results of the phase I CAPRA study of IT CVA21 and Pembrolizumab therapy, also in advanced melanoma, showed an ORR of 73% with regression of injected and non-injected lesions (86), the study has completed although final results have not yet been published. The phase I CANON trial of intra-vesical CVA21 in NMIBC also showed transition of the TME to an inflamed phenotype along with upregulation of immune checkpoints such as PD-L1 and LAG-3 (87).

The modified vaccinia poxvirus JX-594 (Pexa-Vec) is also under investigation in combination with ICPI therapy. A recent phase Ib study in patients with renal cancer reported evidence of treatment response in combination with Cemiplimab therapy. The first phase of this trial involved IV oVV treatment, however the second phase will evaluate localised IT therapy (NCT03294083). A further study recruiting patients with advanced solid tumours for combined IT JX-594 with Ipilimumab is ongoing (NCT02977156).

Intratumoural injection of an oncolytic Vesicular Stomatitis Virus (oVSV) construct VSV-hIFNbetasodium iodide symporter is currently being tested in combination with avelumab in advanced solid tumours (NCT02923466). The oncolytic poliovirus, PVSRIPO, is also being tested in phase I trials in combination with nivolumab in PD-1-refractory melanoma (NCT0412759) and atezolizumab in glioma (NCT03973879). All trials are currently recruiting.

## Radiotherapy

Evidence of involvement of the immune system in the anti-tumour effect of radiotherapy has accumulated over many decades. Radiotherapy-induced regression of tumour lesions distant from the radiation field was first described almost 70 years ago and termed the “abscopal” effect (88). Over the years, this rare phenomenon has been reported in several malignancies (89, 90), while in mice, the role of T cells in controlling tumour growth following radiotherapy has been described more recently (91). These findings have spurred a growing field of research into elucidating the determinants of radiation-induced immune responses as well as the prospect of boosting the abscopal effect with immunostimulatory agents, although whether, or not, the abscopal designation truly applies when a systemic immunotherapy is part of treatment remains a moot point.

Radiotherapy has traditionally been used to treat cancer by utilizing the selective inability of cancer cells to repair DNA damage. When radiotherapy is used as an immune adjuvant, the aim is to transform the tumour into an individualized *in situ* vaccine. This process requires increasing the antigenicity as well as the adjuvanticity of the targeted tumour which is highly dependent on firstly the mode of cell death that the irradiated tumour cells undergo, secondly which molecular signalling pathways are induced and thirdly which DAMPs are released in the TME.

### Radiation-Induced Tumour Antigenicity

Antigenicity is increased by inducing exposure and presentation of mutation-associated tumour neoantigens, which are the key targets for a T cell-mediated anti-tumour immune response, and which correlate with response to ICPI (92). Radiotherapy has been shown to promote an acute transcriptional programme including genes associated with DNA damage and repair, many of which are frequently mutated in tumours (93). Further, radiotherapy increases the peptide pool through augmented protein degradation and mTOR-regulated translation (27). When combined with increased MHC class I expression, this results in more antigenic peptides being presented for recognition by the host immune cells and enhanced TCR diversity (34). Indeed, Lhuillier and Lussier showed that irradiation upregulates genes harboring immunogenic mutations, resulting in selective elimination of irradiated tumour cells by neoantigen-specific CD8 T cells in the 4T1 mouse breast cancer model and KP mouse sarcoma model respectively (30, 94). *In vivo* focal irradiation of 4T1 tumours was shown to broaden the TCR repertoire with expansion of T cell clones driven by anti-CTLA-4 checkpoint inhibition (31). Importantly, in a patient with metastatic NSCLC who experienced a complete response to radiotherapy and ipilimumab, Formenti et al. detected clonal expansion of an immunogenic antigen derived from a gene upregulated by radiation (29).

### Radiation-Induced Tumour Adjuvanticity

DNA damage induced by ionizing radiation causes accumulation of double-stranded DNA (dsDNA) in the cytosol, as well as micronuclei formation. This dsDNA is recognized by cyclic GMP-AMP synthase (cGAS) (95) and subsequently activates stimulator of interferon genes (STING), thus triggering the transcription of Type I IFNs (96). A main role of IFNs in anti-tumour immunity is to recruit DCs (97) and facilitate their maturation and migration to tumour-draining lymph nodes allowing for cross-priming of naïve T cells (98). The resulting activation and bridging of innate and adaptive immune cell responses ultimately promote proliferation and activation of antigen-specific anti-tumour T cells.

ICD is defined by induction of certain DAMPs, all of which are induced by radiotherapy (99) resulting in DC activation in a dose and fractionation-dependent manner (100). The importance of HMGB1 release from irradiated tumour cells for effective radiation-induced tumour response was exemplified by Apetoh and colleagues in two studies reporting dependency of TLR-4 signalling for efficient antigen presentation by DCs and tumour susceptibility to radiotherapy in mice and humans (26, 101). Further, increased translocation of calreticulin in human breast, prostate and lung cancer cells following radiotherapy was shown to increase their sensitivity to CD8 T cell lysis (43). Conversely, radiotherapy was shown to downregulate CD47 in head and neck tumours, counteracting its suppressive effect on DC phagocytosis and resulting in pronounced radiation-induced anti-tumour effect (102).

### Radiotherapy and ICPI Combinations – Pre-Clinical Studies

As evidence mounts of the ability of radiotherapy to alter the immune composition of the TME, there are increasing efforts to implement radiotherapy as an adjuvant to ICPI in patients that are unresponsive to immunotherapy alone.

Radiotherapy (RT) has been shown to promote PD-1/PD-L1-mediated immune resistance, setting the stage for potential synergistic effects. PD-L1 surface expression was shown to be elevated on tumour cells following radiotherapy, which has been attributed to IFNg release from tumour-infiltrating lymphocytes (TILs) (103). Tumour-infiltrating T cells have also exhibited increased expression of PD-1 and 4-1BB following *ex vivo* irradiation of colon- and gastric cancer tumour samples (104). Indeed, PD-1/PD-L1 blockade administered concomitantly with hypofractionated radiotherapy improved tumour control, compared to radiotherapy or ICPI alone, and generated sustained CD8 T cell responses and immunological memory (103) while simultaneously reducing immune suppression by myeloid-derived suppressor cells (MDSCs) (105).

Further, radiotherapy has been shown to potentiate the anti-tumour effect of CTLA-4 blockade in a CD8 T cell-dependent manner in the aggressive and poorly immunogenic breast cancer model 4T1 (31, 33, 106, 107). In this model, radiotherapy was shown to stabilize the immune synapse when CD8 T cells engaged natural killer cell group 2D (NKG2D) with its ligand retinoic acid inducible 1 (Rae-1) on target tumour cells (33). Radiotherapy was also shown to promote T cell recruitment and tumour infiltration by increasing production of the chemokine CXCL16 (107). An increase in the TCR repertoire was demonstrated following RT, with proliferation when RT was combined with anti-CTLA-4 therapy. This was in contrast to single-agent anti-CTLA-4 therapy, which led to fewer T cell clones (31). Similarly, in a melanoma mouse model, CTLA-4 blockade cooperated with radiotherapy to increase the CD8 effector T cell to Treg ratio and diversify the T cell receptor (TCR) repertoire resulting in therapeutic synergy. The anti-tumour effect was further improved by addition of PD-L1 blockade to boost clonal expansion and offset T cell exhaustion (28).

Recently, in the poorly immunogenic, ICPI-refractory KP mouse sarcoma model, which has low mutational status, Lussier et al. reported that low-dose irradiation of KP cells induced immunogenic mutations generating neo-antigens sufficient to convey T cell-mediated protection against the parental cell line *in vivo* when combined with anti-CLTA-4 and anti-PD-1 treatment (94).

### Clinical Translation

Robust pre-clinical evidence has meant that combinations of radiotherapy and ICPI continues to be an area of ever-increasing research interest. There are currently over 500 studies involving clinical testing of these combinations, a number that has greatly increased in recent years (**Table 4**).

**Table 4:** Summary of actively recruiting clinical trials evaluating radiotherapy in combination with immune checkpoint inhibition.

Target	Checkpoint inhibitor	Number of actively recruiting clinical trials
**CTLA-4**	Ipilimumab	51
**PD-1**	Nivolumab	138
	Pembrolizumab	161
	Cemiplimab	6
	Dostarlimab	3
**PDL-1**	Atezolizumab	59
	Durvalumab	115
	Avelumab	23

Several retrospective studies have evaluated the potential benefit of irradiation prior to checkpoint inhibition. Knispel et al. recently reported results of a multi-centre retrospective study of 835 patients with metastatic melanoma receiving anti-CTLA-4 or anti-PD-1 therapy with or without previous radiotherapy for unresectable metastases (108). No evidence of benefit was seen with preceding radiation therapy. In contrast, retrospective analysis of the KEYNOTE-001 phase I trial of NSCLC patients treated with Pembrolizumab showed an improvement in PFS and OS in patients who had previously been treated with radiotherapy (109). Neither study reported an increased risk of adverse events with combination therapy.

A non-exhaustive selection of prospective clinical studies evaluating RT/ICPI combinations is summarised in **Table 5**. Formenti et al. investigated the mechanisms behind response to combination anti-CTLA-4 and radiotherapy in treatment-refractory NSCLC patients in a phase I/II study (NCT02221739). Evidence of response was seen in 33% of evaluable patients, with 2 complete responses. There was no association seen between CD8 T cell infiltration or PD-L1 expression and response, however RT-induced IFNb secretion and sustained TCR clonal expansion was associated with an abscopal response (29). Conversely, a recent phase I study evaluating RT in combination with anti-CTLA-4 in metastatic melanoma showed CD8 infiltration to be significantly correlated with PFS (NCT01557114) (110). McBride et al. also evaluated the mechanics of the abscopal effect in a phase II study of Nivolumab with or without SBRT in metastatic HNSCC. No statistically significant differences were seen between treatment groups, with no evidence of abscopal effects (NCT02684253) (111). The combination of Pembrolizumab and RT in the definitive setting in HNSCC is also being evaluated, a phase II study comparing ICPI therapy with conventional chemoradiotherapy is currently recruiting (NCT03383094).

**Table 5:** A non-exhaustive representative summary of key clinical trials evaluating radiotherapy in combination with immune checkpoint inhibition.

NCT number	Combination	Study design	**Findings**
**NCT02125461**	Sequential Durvalumab after concurrent chemoradiotherapy (PACIFIC trial)	Phase 3, stage III unresectable NSCLC	Median PFS 16.8 months (Durvalumab) vs 5.6 months (placebo)
**NCT02444741**	50 Gy in 5 fractions SBRT + concurrent Pembrolizumab	Phase 1/2, metastatic NSCLC	Improved ORR, did not reach statistical significance
**NCT02492568**	24 Gy in 3 fractions + sequential Pembrolizumab	Phase 2, metastatic NSCLC	Improved ORR, did not reach statistical significance
**NCT02904954**	24 Gy in 3 fractions SBRT+ concurrent Durvalumab prior to surgical resection	Phase 2, stage I, II, IIa NSCLC, neo-adjuvant	Significantly higher major pathological response rate with combination treatment (53.3%) *vs* single agent Durvalumab (6/7%)
**NCT02221739**	30 Gy in 5 fractions (later 28.5 Gy in 3 fractions) RT + concurrent Ipilimumab	Phase 1/2, metastatic NSCLC.	Evidence of response in 33% of evaluable patients.
**NCT01557114**	9, 15, 18 or 24 Gy in 3 fractions RT + concurrent Ipilimumab	Phase 1, advanced melanoma	31% ORR, increased CD8+ T cells associated with improved PFS
**NCT02684253**	27 Gy in 3 fractions + concurrent Nivolumab	Phase 2, HNSCC	No improvement in response and no evidence of abscopal effect
**NCT02730130**	30 Gy in 5 fractions + concurrent Pembrolizumab	Phase 2, TNBC	ORR 17.6%, 3/17 CR
**NCT03051672**	20 Gy in 5 fractions + Pembrolizumab 2-7 days prior then every 21 days	Phase 2, metastatic hormone receptor positive, HER-2 negative breast cancer	No objective responses, median OS 2.9 months
**NCT02311361**	8 Gy single fraction or 25 Gy in 5 fractions + Durvalumab/Tremelimumab/dual ICPI	Phase 1/2, PDAC	ORR 5.1%, PFS between 0.9 and 9 months depending on treatment cohort

Several key studies have evaluated combinations of RT and checkpoint blockade in breast cancer, a site where RT presents a cornerstone of treatment and where ICPI therapy has shown limited efficacy to date. Phase III trials have supported the approval of ICPI therapy in PD-L1-positive TNBC patients in combination with chemotherapy, and subsequent trials of combination ICPI/RT have yielded mixed results. Triple-negative breast cancer is classically seen to be poorly immunogenic, however has a high mutational burden with significantly higher PD-L1 expression than other sub-types (112). A phase II study of RT and Pembrolizumab in metastatic TNBC patients not selected for PD-L1 expression showed that treatment was well tolerated with some evidence of clinical activity in this poor-prognosis group (17.6% ORR). The study reported 3 complete responses and evidence of response outside the radiation field (NCT02730130) (113). In contrast, Barroso-Sousa et al. reported negative results of pembrolizumab and palliative radiotherapy in a small 8 patient study of heavily pre-treated hormone receptor positive metastatic breast cancer patients. No objective responses were seen, and the median overall survival was 2.9 months (NCT03051672) (114). Trials of additional combinations are planned, for example a phase 2 study combining Atezolizumab, radiotherapy and the TLR-7/8 agonist BDB001 is currently recruiting patients with PD-1/PD-L1-refractory TNBC (NCT03915678). Three trials combining RT/ICPI and Parp inhibitors are also planned in PD-L1-negative or ICPI refractory metastatic TNBC (NCT04690855, NCT04683679).

As previously discussed, pancreatic cancer is notoriously immune-excluded and ICPI-refractory. A phase I study recently evaluated the safety of stereotactic body radiation therapy (SBRT) and Durvalumab or Tremelimumab treatment. No dose-limiting toxicities were seen with combination therapy, and 2/39 patients had a partial response with an ORR of 5.1% and PFS between 0.9 and 9 months depending on treatment cohort (NCT02311361) (115).

The phase III PACIFIC trial showed that Durvalumab therapy significantly improved survival compared to standard of care concurrent chemo-radiotherapy in patients with locally advanced stage III unresectable NSCLC (NCT02125461). Antonia et al. reported a pronounced benefit in PFS with Durvalumab treatment compared with placebo (16.8 months vs 5.6 months respectively) (116), highlighting the potential for upfront combination therapy in the definitive management of locally-advanced disease despite the mixed results discussed above. These findings represent a pivotal milestone for the clinical implementation of RT/ICPI combination therapy, and the subsequent PACIFIC-4 trial has extended this combination to early-stage NSCLC in stage I/II node negative disease.

Conversely, in locally-advanced HNSCC, the phase 3 JAVELIN-100 trial evaluating avelumab in combination with standard-of-care chemoradiotherapy failed to meet its primary endpoint of prolonged PFS. Of relevance, subgroup analysis showed the only PFS benefit to be in patients with tumours expressing high levels of PD-L1 (>25%) at baseline, and further research is needed to evaluate the barriers to effective therapy in “cold” tumours (117).

In the metastatic setting, the PEMBRO-RT (phase 2, NCT02492568) (118) and MDACC (119) (phase 1/2, NCT02444741) trials both noted a treatment benefit with combination pembrolizumab/RT in NSCLC. This did not reach statistical significance overall due to a small sample size, however significance was noted in both studies in an exploratory analysis of the sub-group of patients with tumours expressing low levels of PD-L1 suggesting a potential benefit in “cold” tumours. A recent pooled analysis of these two studies showed a significant improvement in outcomes with the addition of RT when compared to single-agent Pembrolizumab, with an OS of 19.2 months vs 8.7 months respectively (120).

A further treatment setting under evaluation is neo-adjuvant treatment of patients with early-stage disease. A recent phase II study of Durvalumab and SBRT therapy in NSCLC (NCT02904954) showed a significant increase in major pathological response rates with combination therapy when compared to single agent Durvalumab (53.3% and 6.7% respectively), validating the strategy for a larger trial (121). This setting has also been evaluated in breast cancer. Pre-operative RT and Pembrolizumab prior to standard-of-care in patients with TNBC was shown to be well tolerated in published interim results, with a pCR or 67%. Of note, baseline TIL count of >10% was shown to corellate with complete response, but not change in TIL over treatment (122). Finally, encouraging results were seen in a recent phase Ib trial of neo-adjuvant SBRT and anti-PD-1 therapy in HNSCC (NCT03247712), where the combination was seen to be well tolerated with a high rate of major pathological response (86%) (123).

## Other Localised Therapies

Other strategies aimed at TME manipulation towards a more inflamed phenotype are also in clinical development. These include locally-delivered immune-adjuvants, non-viral oncolytics, and physical thermal therapies such as high intensity focused ultrasound (HIFU). A non-exhaustive selection of ongoing clinical studies are summarised in **Table 6**.

**Table 6:** A non-exhaustive summary of ongoing clinical trials evaluating other localised therapies in combination with immune checkpoint inhibition.

Agent and NCT number	Combination	Study design	Status
**Rose Bengal Disodium (PV-10)**			
NCT02557321	PV-10 + Pembrolizumab	Phase 1, ICPI-refractory advanced melanoma	Recruiting
**TLR agonists**			
NCT03865082	Tilsotolimod (TLR-9 agonist) + Ipilimumab and Nivolumab	Phase 2, solid tumours	Recruiting
NCT04633278	CMP-001 (TLR-9 agonist) + Pembrolizumab	Phase 2, HNSCC	Recruiting
NCT03435640	NKTR-262 (TLR-7/8 agonist) + Nivolumab/pegylated-IL2	Phase 1/2, advanced solid tumours	Active, not recruiting
NCT03301896	LHC-165 (TLR-7 agonist) + PDR001 (anti-PD1)	Phase 1, advanced solid tumours	Active, not recruiting
NCT03317158	BCG + Durvalumab + RT	Phase 1/2, NMIBC	Recruiting
**STING agonists**			
NCT03010176	MK-1454 + Pembrolizumab	Phase 1, advanced solid tumours	Active, not recruiting
NCT04220866	MK-1454 + Pembrolizumab	Phase 2, HNSCC	Active, not recruiting
NCT03937141	ADU-S100 + Pembrolizumab	Phase 2, HNSCC	Active, not recruiting
**Oncolytic Peptides**			
NCT04796194	LTX-315 + Pembrolizumab or Ipilimumab	Phase 2, advanced solid tumours	Recruiting
**Thermal treatments**			
NCT03237572	HIFU + Pembrolizumab	Phase 1, metastatic breast cancer	Recruiting

### PV-10

Rose Bengal disodium is a small-molecule analogue of the commonly-used conjunctival dye fluorescein and is under clinical evaluation in its injectable form PV10 as a cancer immunotherapy. Intralesional PV-10 has been shown selectively to accumulate in lysosomes within tumour cells, leading to immunogenic cell death, PAMP, DAMP and TAA release, and an antigen-specific anti-cancer T cell response. Pre-clinical synergy has been shown with IT PV-10 and anti-PD-L1 therapy, with the initiation of a CD8 T cell-dependent anti-tumour immune response and depletion of Treg (124). A phase Ib trial combining PV-10 with pembrolizumab met its primary endpoint of safety in advanced melanoma and led to a complete response (CR) in 9% with partial response in 57% - translational correlative T cell data are awaited (125). Two expansion cohorts are currently recruiting patients with checkpoint-inhibitor-refractory melanoma (NCT02557321).

### Toll-Like Receptor Agonists

Toll-like receptors (TLRs) are a family of PRRs that are most commonly found on DCs and macrophages, but also on T cells and tumour tissue. They play a key role in the innate and adaptive immune response, recognising potentially harmful PAMPs and DAMPs including microbial nucleic acids and TAA and triggering apoptosis and immune cell maturation and recruitment.

Bacillus Calmette Guerin (BCG) is a live attenuated strain of Mycobacterium Bovis, a potent agonist of TLR-2 and 4 that has been routinely used in the treatment of bladder cancer for decades. Schmidt et al. showed intra-tumoural injection of the TLR-9 agonist lefitolimod led to remodelling of the TME to a “hot” phenotype in mouse CRC models – with CD8 T cell influx, an increase in the CD8:Treg ratio and a greater proportion of M1-polarised macrophages (126). Enhanced therapeutic effect was seen in combination with anti-PD-1 therapy (127).

Several clinical trials are ongoing evaluating TLR agonist/ICPI combinations. The phase I/II ILLUMINATE-204 multi-centre study evaluated intra-tumoural TLR-9 agonist (tilsotolimod) therapy in combination with Ipilimumab in PD-1 refractory metastatic melanoma (NCT02644967). Responses were seen in local and distant lesions, with a 22.4% ORR (2 complete responses) and a 21-month median OS. Tumour biopsies showed evidence of an IFNa inflammatory gene signature and expansion of CD8 T cell clones (128). The subsequent ILLUMINATE-301 trial failed to achieve its primary end point, with no significant improvement in ORR over Ipilimumab alone (NCT03445533) (129). A further phase II study is ongoing recruiting patients with microsatellite stable (MSS) colorectal cancer (CRC) for intra-tumoural tilsotolimod in combination with ipilimumab (ILLUMINATE-206, NCT03865082). Early results showed the combination to be generally well tolerated with some evidence of response in injected and non-injected lesions.

Milhem et al. (130) reported early results of an ongoing phase 1b trial of a further TLR-9 agonist (CMP-001) and pembrolizumab in patients with PD-1-refractory melanoma. A best ORR of 23.5% was seen with a median duration of response of 19.9 months (NCT02680184). CMP-001 was also evaluated in a recently completed phase I study in combination with atezolizumab in PD-1-resistant NSCLC with or without radiation therapy (NCT03438318). CMP-001 was delivered SC (weeks 1 and 2) then IT (weeks 3-5) into visceral lesions. Treatment had a tolerable safety profile and stable disease was seen in a subset of patients. However, enrolment was stopped after Stage 1 due to no objective responses CMP-001 is also being evaluated in a Phase II trial in combination with Pembrolizumab in HNSCC (NCT04633278). Preliminary results of a Phase Ib study of TLR-7/8 agonist NKTR-262 in combination with Nivolumab and pegylated IL2 in advanced solid tumours (NCT03435640) showed enhanced immune infiltration and early evidence of clinical activity (131).

Other ongoing clinical trials include the IT TLR-7 agonist LHC165 in combination with anti-PD-1 in patients with advanced solid tumours (NCT03301896), the TLR-8 agonist motolimod in combination with anti-PD-1 agent nivolumab in HNSCC (Phase 1, NCT03906526) and BCG in combination with Durvalumab +/- RT in NMIBC (NCT03317158).

### STING Agonists

The adaptor protein, STING, is a critical component of the previously discussed cGAS/STING pathway and acts as a bridge between innate and adaptive immunity. Cytosolic microbial or tumour-derived DNA is sensed by the PRR, cGAS, which undergoes conformational changes to catalyse ATP and GTP into the cyclic di-nucleodide (CDN) cGAMP. STING is activated on binding with cGAMP or other CDNs, leading to stimulation of a type 1 IFN response, immune cell recruitment, promotion of DC maturation and priming of antigen-specific immunity.

Most STING agonists in clinical development are human, bacterially-derived or synthetic CDNs mimicking cGAMP. As STING is located intracellularly on the ER, any agonist must penetrate the cell membrane, leading to low bioavailability of natural CDNs which are hydrophilic, electronegative and large in size. Localised delivery *via* intratumoural injection therefore provides a mechanism to enable therapeutic dosing within the TME, although emerging novel agents such as non-nucleotide small-molecule systemic STING agonists are in development for intravenous or oral administration (132).

Preclinical evidence provides rationale for the combination of STING agonism and ICPI therapy. In a poorly-immunogenic mouse sarcoma model, STING deficiency was shown to limit response to dual ICPI therapy highlighting an element of dependence on STING-mediated immunity (22). Ager et al. demonstrated that intratumoural injection of a STING agonist in a poorly-immunogenic bi-flank model of TRAMP-C2 mouse prostate cancer led to regression of injected but not non-injected tumours. Addition of checkpoint therapy led to synergistic effects in both injected and non-injected tumours, with an influx of CD8 effector cells, macrophage reprogramming and an increase in CD8:Treg ratio (133).

Combination treatment with STING agonism and anti-PD-1 therapy was shown to enhance therapeutic effects in the T cell-inflamed MOC1 mouse model of HNSCC. In contrast, the non-T cell-inflamed MOC2 model, used to represent “cold” tumours in work by Moore et al, did not respond to either single agent STING agonist or combination ICPI therapy. In these tumours, STING agonism induced a type I IFN response but did not result in CD8 TIL recruitment highlighting the inter-tumoural complexity in TME modification (134). Enhanced efficacy was seen with a combination of intraperitoneal STING agonist in combination with conventional carboplatin chemotherapy and anti-PD-1 checkpoint blockade in a model of high-grade serous ovarian cancer, a notoriously “cold” tumour site. STING agonism was shown significantly to enhance IFN production, the infiltration of activated PD-1-expressing CD8 T cells and MHCII expression in tumour-bearing mice (135).

A number of clinical trials evaluating STING agonism and ICPI combinations are currently ongoing. A first in human phase I study of the STING agonist MK-1454 in combination with Pembrolizumab in advanced solid tumours or lymphoma (NCT03010176) reported an encouraging safety profile and early evidence of efficacy (136). Phase II studies evaluating STING agonists MK-1454 (NCT04220866) and ADU-S100 (NCT03937141) in combination with Pembrolizumab in HNSCC are currently active.

### Melphalan

Melphalan is a nitrogen mustard alkylating chemotherapeutic agent that has been widely used in cancer therapy. While systemic therapy is known to cause lymphopaenia, localised therapy has been shown to enhance immune cell infiltration and antigen presentation through the initiation of apoptotic ICD, while minimising systemic side effects. Ariyan et al. showed synergy between local melphalan delivered *via* isolated limb perfusion and systemic anti-CTLA-4 therapy, with remodelling of the TME to an inflamed phenotype. This was translated into a phase II clinical trial, where combination therapy was shown to improve PFS with 62% complete responses and median PFS not reached. However, this did not reach significance over either treatment alone in the study of 26 patients (137).

### Oncolytic Peptides

Designed to mimic natural antimicrobial peptides, oncolytic peptides (OPs) are short polypeptides with a net positive charge and a large proportion of hydrophobic amino acid residues (138). This allows them selectively to enter through negatively-charged phospholipid membranes, which are preferentially found in cancer cells due to higher phosphatidylserine exposure (139). Oncolytic peptides LTX-315/401 (140) and RT53 (141) have been shown to trigger ICD and DAMP release (ATP, HMGB1 and Calreticulin), as well as IFN I secretion, in melanoma and fibrosarcoma models; this was associated with local immune infiltration and tumour regression. In a mouse ovarian cancer model, local administration of the gonadotropin-releasing hormone receptor (GNRHR)-targeted peptide EP-100 was combined with anti-PD-L1-generating NK cell, DC and CD8 T cell tumour infiltration. In a process dependent on interleukin (IL)-33, T regs were simultaneously depleted. In mouse models of fibrosarcoma (MCA205) and lung carcinoma (TC-1), I.T. injections of LTX-315 (142)and LTX-401 (143) in combination with anti-PD-1 or anti-CTLA-4 promoted immune-dependent control of injected and abscopal (non-injected) tumour lesions.

LTX-315 is currently being explored in a phase I trial including patients with transdermally-accessible tumours in combination with pembrolizumab (NCT04796194).

### Thermal and Ultrasound Based Treatments

High-intensity focused ultrasound (HIFU) is a non-invasive thermal modality, primarily used to treat solid tumours in cancer patients who are poor candidates for surgery and radiotherapy. HIFU was shown to induce ICD of human cancer cells promoting generation of DAMPs as well as cytokines that could polarize macrophages from a suppressive M2- to an anti-tumour M1-phenotype (144, 145). In immunocompetent mice, HIFU boosted DC infiltration in treated tumours and promoted CD8 T cell cytotoxicity (146). The documented effects of HIFU on DC recruitment, macrophage polarization and stromal dissociation indicate that HIFU treatment could skew the TME towards immune activation and possibly potentiate the effect of ICPI or immune agonists to generate systemic and tumour-specific immune responses. An ongoing phase 1 study is currently evaluating HIFU in combination with Pembrolizumab in metastatic breast cancer (NCT03237572).

Radiofrequency ablation (RFA) and microwave ablation (MWA) uses needle-like electrode probes to deliver radiofrequency and electromagnetic waves respectively, generating oscillation and subsequent heating of the tumour tissue. In two mouse models of breast (4T1) and colon (CT26) cancer, MWA of primary tumours followed by combined anti-PD-1 and anti-CTLA-4 treatment resulted in prolonged survival compared with MWA or ICPIs alone. MWA + ICPI treatment was also associated with increased frequencies of CD8 T cells in treated tumours and peripheral blood as well as increased plasma levels of IFNγ (147). Neo-adjuvant RFA of NSCLC tumours showed prominent CD8 and CD4 T cell infiltration in the peripheral regions of RFA-treated tumours as well as increased frequency of pro-inflammatory BDCA-3+ DCs in peripheral blood suggesting systemic immune activation (148). A retrospective study of colorectal cancer patients who had received preoperative RFA to liver metastases showed increased number of CD4 and CD8 TILs and increased PD-L1 expression in the resected primary tumours. An RFA-induced transient abscopal immune activation and PD-L1 induction was observed in a CT26 mouse tumour model with combined RFA and anti-PD-1 antibody treatment showing synergistic T-cell mediated systemic immunity (149).

Photothermal therapy (PTT) works by administering optically-absorbent nanoparticles which, when activated by near-infrared light, generates heat and localised thermal damage (18). PTT, in combination with a TLR-7 agonist and anti-CLTA-4 antibodies, induced abscopal effects in an orthotopic 4T1 breast cancer model (150). A recent case report describes a treatment-refractory HNSCC patient achieving a complete and sustained tumour response to photodynamic therapy (PDT) with anti-PD-1 antibody (151).

## Discussion and Future Directions

Despite huge advances in recent years, ICPI therapy remains largely ineffective in the treatment of immunologically cold tumours. Although synergistic effects have been widely demonstrated between localised immune-modulatory therapies and ICPI, clinical response rates remain suboptimal. The mechanisms of response and resistance are highly complex, and it is likely that no single therapy will overcome tumour-mediated immunosuppression across multiple tumour histotypes. Multi-targeted combinations are likely to represent the future of immunotherapeutic strategies, and an overwhelming number of combinations are currently in clinical development.

Localised therapy combinations have been shown to have synergistic effects in some studies. RT has been shown to synergise with OV and ICPI, for example a CTLA-4 armed oNDV was shown to enhance sensitisation of melanoma cells to radiation (152). Oba et al. also demonstrated efficacy of *in situ* immune modulation using sequentially delivered local therapies – RT, DC recruitment agent Fms-like tyrosine kinase 3 ligand (Flt3L) and TLR-3/CD40 stimulation - in overcoming checkpoint resistance in an immune-excluded mouse melanoma model (153). A further recent clinical study provided evidence that sequential oADV/HSV-TK, SBRT and anti-PD-1 was able to restore ICPI sensitivity in NSCLC patients, with a 64.2% clinical benefit rate (CBR) in patients that had received prior ICPI therapy (154). This highlights the potential benefit of a multi-targeted approach, even within localised therapies, and further triple combinations are emerging, such as STING or TLR agonists in combination with RT and ICPI.

Systemic targeted agents may also enhance treatment effects, and one novel strategy is the use of agents that target cellular DNA damage repair (DDR) pathways. An effective anti-cancer immune response is dependent on the formation of tumour neoantigens regardless of TME immunogenicity, a process that may be a key limiting factor in ICPI efficacy in cold tumours, due to a low mutational load. PARP inhibitors (PARPi) lead to un-repaired DNA damage which triggers a cascade of immunogenic events including enhanced PD-L1 expression on tumour cells, immune cell infiltration and TAA formation (155), providing rationale for combination treatment in cold tumours. PARPi was seen to enhance OV-mediated oncolysis in a model of anaplastic thyroid cancer (156). Treatment was also shown to enhance radiosensitivity, with PARPi/RT leading to chemokine secretion, immune infiltration and upregulation of PD-1/PD-L1 (157). Clinical trials evaluating triple combination with PARPi/RT/ICPI are ongoing (for example NCT04837209 and NCT04926324). The ataxia telangiectasia and Rad3-related (ATR) kinase is also integral to DDR pathways. Treatment with an ATR inhibitor was shown to synergise with radiotherapy and checkpoint inhibition in a pre-clinical model of liver cancer (158).

Combinations involving co-stimulatory agonists such as 4-1BB or CD40-L are also in clinical development. A recent study in an orthotopic mouse model of pancreatic ductal adenocarcinoma (PDA) revealed that an agonist targeting the co-stimulatory receptor CD40 synergized with radiotherapy to promote systemic tumour-targeted immune responses in combination with dual ICB (159). In mouse models of colorectal (MC38), melanoma (B16-OVA) and breast (4T1) cancer, radiotherapy administered concomitantly with a 4-1BB agonist resulted in local and abscopal anti-tumour immune responses and prolonged survival, which was dependent on CD8 T cells and conventional type I dendritic cells (cDC1a) (104). Such combinations have also been investigated in the context of oncolytic virotherapy, where the viral backbone presents an opportunity for delivery of these agents limiting toxicity. For example, Coffin et al. are currently testing an oHSV armed with anti-CTLA-4, a 4-1BB agonist and CD40-L in early phase clinical trials (68).

Novel checkpoints such as TIGIT, TIM-3, LAG3 (160) or other inhibitory targets such as the CEACAM proteins (161) are also under investigation, as are other triple combinations involving alternate immunotherapies such as CAR-T cells (162) or Bi-specific T cell engagers (BiTE) (163). As more is known about the effects of T cell modulatory therapies such as ICPI on the non T-cell constituents of the TME, the understanding and effective application of future ICPI combinations is likely to increase.

For example, the PD-1 pathway also regulates NK cells, B cells and macrophages, and evidence for the impact of the diverse cellular constituents of the TME on response to ICPI continues to accumulate. Indeed, a study of ICPI therapy in melanoma showed enrichment of B cell signatures in responding patients (164), and conditional knockout of novel checkpoint TIM-3 on DCs was shown to lead to inflammasome activation and anti-tumour immunity, an effect that was not seen with TIM-3 deletion on CD4 or CD8 Tcells (165).

Selective deletion of PD-1 in myeloid cells has also recently been shown to induce a more effective anti-cancer immune response than ablation on T cells (166), and other innate components such as Group 2 innate lymphoid cells (ILC2s) are also emerging as critical elements of checkpoint-mediated anti-cancer immunity (167). Understanding the complexities of these relationships will be essential in deciphering mechanisms of response, and identifying targets to overcome resistance.

As more complex combinations move into the spotlight, the question of optimal dose delivery and scheduling becomes of paramount importance both with regards to efficacy and toxicty. There is a rationale for checkpoint blockade as a priming agent before radiotherapy, and equally for OV administered prior to ICPI to prime for an effective anti-cancer immune response. Conversely, concurrent administration may enable maxiumum synergy, and sequential ICPI delivered after localised therapy may be the most effective way of sustaining immunity and overcoming T cell exhaustion. This is currently under investigation, and ongoing trials will begin to shed light on optimal scheduling. For example, a study evaluating priming vs concurrent atezolizumab in combination with conventional chemoradiotherapy in cervical cancer has recently finished recruiting (NCT03738228). A study combining an oADV with anti-PD-L1 agent Durvalumab is also currently recruiting and will evaluate concomitant *vs* sequential treatment (NCT03799744).

In the context of radiotherapy, the differential effects of dose-fractionation on immunogenicity are also largely unknown. Demaria et al. reported that RT doses above 12-18 Gy induced the Trex1 exonuclease and attenuated any immunogenic effects, while repeated lower doses led to IFN production, DC recruitment and immune-cell priming (23), and fractionated regimens have been preferred in some studies. For example, Dewan et al. reported that fractionated but not single dose (20 Gy) radiotherapy induced an abscopal effect in a murine model when combined with an anti-CTLA-4 antibody (168). In comparison, some studies report hypofractionated doses are more immunogenic, with a 15 Gy single fraction resulting in greater tumour control and increased activation and infiltration of antitumor T cells compared to 3 Gy x 5 in a B16/OVA murine model of melanoma (169). Further questions include treatment volume, especially when considering the immune effects of lymph node irradiation, which has been shown to attenuate adaptive anti-cancer immunity by altering CD8 T cell trafficking. Further evaluation may have an impact on radiotherapy target volumes of the future (170).

For OV treatment, a key limitation to optimal delivery is that many typically “cold” tumour sites such as brain, pancreatic and ovarian, do not have easily accessible lesions for repesated injection. Viral infectivity is highly heterogenous, and the barrier of anti-viral immunity, either from prior infection, vaccination or neutralizing antibodies (nAb) secondary to OV treatment, also hinders viral replication and therefore efficacy following systemic delivery. Novel methods of viral encapsulation to overcome the barrier of anti-viral immunity may provide a potential method of enhancing anti-tumour effects, for example, Francini et al. published evidence of ablation of nAb binding without viral inactivation using a new class of coating polymers (171).

There is much still to learn in order to overcome the lack of ICPI efficacy in cold tumours and to maximise the synergistic benefit of localised therapy combinations. However, continued research intended specifically to understand the biological changes occurring in tumours following administration of localised therapies is laying the groundwork for the design of more effective strategies. Checkpoint inhibitors have been proven to provide a widely-applicable method of immune rejuvenation, and are likely to form an integral part of future strategies. With the ability of localised therapies to manipulate the TME and enhance tumour immunogenicity without excessive additive side effects, they remain an attractive addition to the therapeutic armoury, with the potential of rendering more patients responsive to immune checkpoint blockade.

## Author Contributions

EA and EW devised and conceptualized the idea and drafted the manuscript. NS designed the figure. JH, CC, AW, AS, MO, AM, and KH have contributed to the writing of the manuscript. All authors contributed to the article and approved the submitted version.

## Funding

Supported by Cancer Research UK (CRUK), the Institute of Cancer Research/Royal Marsden Hospital Centre for Translational Immunotherapy and RadNet Radiation Research Network.

## Conflict of Interest

The authors declare that the research was conducted in the absence of any commercial or financial relationships that could be construed as a potential conflict of interest.

## Publisher’s Note

All claims expressed in this article are solely those of the authors and do not necessarily represent those of their affiliated organizations, or those of the publisher, the editors and the reviewers. Any product that may be evaluated in this article, or claim that may be made by its manufacturer, is not guaranteed or endorsed by the publisher.
